# A Review of Central Serous Chorioretinopathy: Clinical Presentation and Management

**DOI:** 10.7759/cureus.27965

**Published:** 2022-08-13

**Authors:** Jerin Varghese, Dipanshu Kesharwani, Shreya Parashar, Prerna Agrawal

**Affiliations:** 1 Ophthalmology, Jawaharlal Nehru Medical College, Datta Meghe Institute of Medical Sciences, Wardha, IND

**Keywords:** retinopathy, retinal detachment, retinal degeneration, choroidal neovascularization, central serous chorioretinopathy

## Abstract

Central serous chorioretinopathy (CSC) may be understood as a disease of the chorioretina with the serous detachment of the neurosensory retina, which is secondary to single or multiple localized defects in retinal pigment epithelium (RPE). CSC is one of the common forms of loss of vision, usually seen in people who do belong to the working-age group. The most common symptoms are blurring of vision, usually unilateral and which is perceived as a scotoma in the center of the field of vision with associated metamorphopsia and micropsia. The risk factor associated with CSC is psychosocial stress, type A personality, pregnancy, and hypercortisolism. Normal vision is often restored within a span of a few months. After around three months, if the resolution of acute CSC did not change or, let us say, in the case of CSC that is chronic, one should consider treatment. In acute CSC, to resolve symptoms, especially in individuals who work in a field where eyesight is of utmost importance, for example, pilots, focal photocoagulation of leaking RPE lesions can be performed. CSC is a prototype cause of serous neuroretinal detachment, which involves the fovea. CSC symptoms reflect the separation between the RPE and the photoreceptors and the bullous distension of the foveal retina. The effect of therapy as such on the long-term outcome of vision visual is not sufficiently documented. The management would largely be dependent on the appropriate diagnosis made based on clinical presentations, and thus it becomes very much necessary to have knowledge about the same and counsel the patient regarding the association between stress and disease pathology. In acute CSC, retinal photocoagulation is successful to a good extent in eliminating or reducing the leakage of RPE and hence it induces resolution of the serous detachment. This review article is made to make sure the reader is updated about the various clinical and management aspects of CSC by providing a comprehensive idea that is obtained from various well-acknowledged databases across the globe on CSC.

## Introduction and background

**Figure 1:**
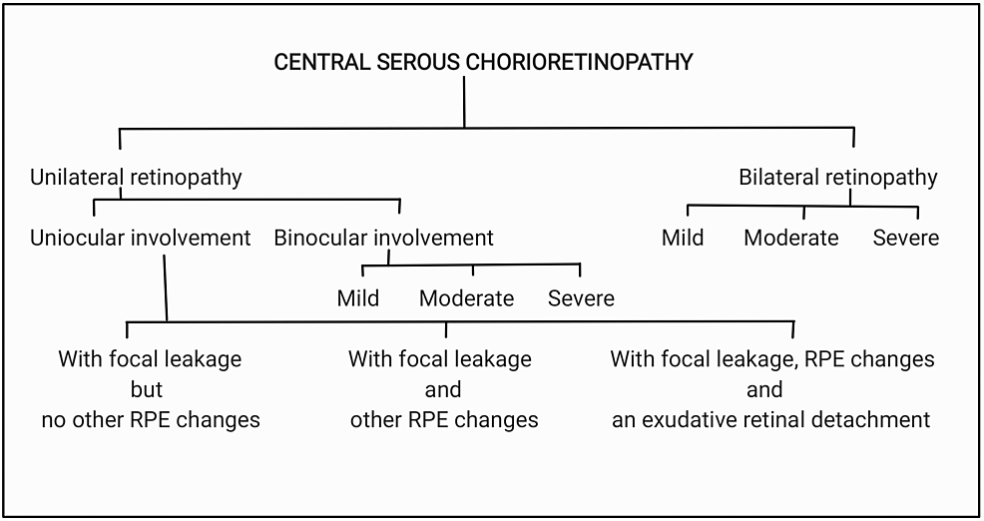
Classification of Central Serous Chorioretinopathy RPE, retinal pigment epithelium.

Central serous chorioretinopathy (CSC) was described for the first time in 1886 by Albrecht von Graefe as a recurrent serous detachment of the macula and named recurrent central retinitis. The other name for chronic CSC is diffuse retinal pigment epitheliopathy. Nearly after 100 years, in 1967, Gass applied the term idiopathic along with central serous choroidopathy. Since then, this disease appears to involve both the choroid and retina, and, thus, the accepted name currently is central serous chorioretinopathy. At the posterior pole of the fundus, there is an accumulation of transparent fluid, which is a characteristic feature of CSC. It predominantly affects the young or middle-aged (25 to 50 years) group, with a male preponderance (male:female ratio = 6:1 ). It is usually unilateral at presentation. Mainly, there are two types of CSC: the classic or typical CSC, which is a more common type seen in younger patients and causes a localized acute detachment of the neurosensory retina at macula with mild-to-moderate visual acuity loss associated with one or more focal leaks which could be appreciated during fluorescein angiography. The other type of CSC has a widespread pigmentation alteration of the retinal pigment epithelium (RPE) mainly related to chronic shallow subretinal fluid (SRF)'s presence. Usually, CSR resolves on its own but may also lead to the portrayal of recurrent episodes, which ultimately end up in a poorer visual prognosis. CSC presents a diverse range of clinical manifestations, which could be evidently understood by the use of multimodal imaging [1]. Time and again, many clinical features were added with respect to CSC, and still, the saga continues based on the findings of newer sophisticated research on the same. On the other hand, there are cases of CSC in which the patient remains asymptomatic over the initial phase of the disease. Many treatment modalities have been implemented to manage CSC, and in the near future, there are hopes that better methods to manage the same will be documented and used. The use of eplerenone has been found to be effective in managing chronic CSC that is therapy-resistant [2]. CSC could broadly be divided into unilateral and bilateral retinopathy (see Figure 1).

CSC is one of those common disease presentations of the eye's posterior segment and frequently leads to mild-to-moderate visual loss. Patients with CSC primarily complained of dark spots (most prominent immediately after awakening) in the visual field's center in one eye with or at times without accompanying metamorphosis. The dark spot can be seen sometimes in the latter part of the day by frequent blinking. It may associate with blurred vision, hypermetropization, micropsia, and reduced contrast sensitivity. CSC can precede migraine in some cases. The hyperopic shift is usually seen in CSC patients. By scanning with the help of laser ophthalmoscopic microperimetry, a 10-100-fold sensitivity reduction within the affected part of the field of vision could be appreciated. Entopic spots reduced by the detachment of serious retina can be made visible by using the pinhole flicker test on the patient. Chronic CSC patients rarely complain of seeing a dark spot as compared to acute CSC patients. The factors that put an individual at risk are anti-psychotic medication, type-A personality, psychological stress, and depression, which in turn would make the scenario complicated by increasing the risk of recurrence. Obstructive sleep apnea, hypertension, and patients on local and systemic corticosteroid therapy are a higher chance of developing the disease [3]. Reports of two or even more cases within the same family also suggest a hereditary predisposition as far as CSC is concerned. There is the presence of a minimal relative afferent pupillary defect in CSC patients. Hemorrhagic detachment of the pigment epithelium has been linked with CSC, according to a study [4].

The long-term prognosis of CSC depends on the degree of extension of pigmentary changes into the macular area [5]. On biomicroscopy, granular material accumulation is seen between the neurosensory retina and the RPE, which increases in relation to the duration of symptoms. Other findings include normal foveal light reflex's absence and yellow foveal xanthophylls' distinct visibility, which is caused by excessive scatter in the thickened area of the detached retina. The role of fluorescent angiography in diagnosis plays an important role in the evolution of the pathophysiology of CSC. It also helps to make a differential diagnosis, notably of subretinal neovascularization, which is also a complication of chronic CSC. The diagnosis can be made without fluorescent angiography as it is diagnosed clinically. The common angiographic finding in fluorescein angiography is the presence of one or more hyperfluorescent leaks in the RPE level (Inkblot appearance). It also demonstrates smokestack configuration in the late phase. Sometimes, a mechanical defect at the margin of the pigment epithelial detachment (PED) may be apparent as a puncture or "blow-out" [6]. Fundus autofluorescence showed confluent, granular hypo-autofluorescence in acute CSC. In chronic-recurrent CSC, irregular and increased hyper-autofluorescence can be seen. Optical coherence tomography (OCT) is an effective method for quantifying neurosensory retinal detachments in cases of CSC. It provides the clinical course's objective measure, thereby reducing the dependence on angiograms [7]. It is specially used for diagnosing chronic cases to explain visual potential to the patient.

The use of indocyanine green angiography (ICGA) to study CSC has extended our knowledge of the disease. Studying the choroidal circulation's abnormalities in CSC by indocyanine green has led to new theories regarding the pathogenesis of CSC. A consistent finding shown in CSC is the choroid's hyperpermeability which appears localized in the inner choroid during ICGA. These areas could be best visualized in the angiogram's mid-phase [8]. Serous detachment in CSC generally resolves spontaneously within three months, with good visual recovery. In some cases, subsequent pigment epithelial scar can be observed, which can be considerably larger than the original leakage point and is characterized by the mixed presentation of hyperfluorescence and hypofluorescence with no signs of dye leakage [9].

Development of recurrences is found in about one-third to half of all the cases, especially in cases of depression and patients with stress. Counseling plays an important role in the management in case of acute CSC. Generally, treatment is required for chronic or recurrent CSC, in an occupation where visual acuity plays an important role, and in the case of monocular patients. Photocoagulation typically shortens the course of the disease and accelerates the resolution of detachment. Generally, a laser is indicated in an area remote from the macula. However, there is no such great effect on the final acuity of vision [10]. Because CSC is a disease that is self-limiting usually, there are possible complications associated with laser treatment. Laser treatment should be avoided in a patient with primary detachment with decreased vision who has a complaint of permanent loss of vision from an untreated detachment of the macula in the fellow eye and also recurrent detachment of macula in the eye which has had an experience of permanent loss of vision as a result of the initial episode. Additionally, laser treatment is required in severe forms of CSC that are known to have a very poor prognosis if left untreated. Laser photocoagulation usually takes about two weeks for anatomical resolution of macular detachment in cases that are uncomplicated, but it may even take approximately six weeks in long-standing detachments with turbid SRF. Complete recovery of vision generally takes twice as that much time.

Photodynamic therapy (PDT) has some advantages over laser photocoagulation in treating CSC. Laser has complications like permanent scotoma and scar formation with permanent foveal damage. On the other hand, PDT does not have clinically visible structural damage, and it usually does not result in permanent scotoma. Recently, PDT has shown promising results in the case of chronic CSC, especially in cases with diffuse decompensation of RPE. Despite long-standing macular detachments of duration that are long, fluid's complete resolution was noted in most of the patients. It is used commonly in those cases where it was considered to be wise to avoid photocoagulation because of the subfoveal or juxta foveal location of the lesion of RPE. The grounds behind such a therapeutic approach are to cause a reduction of the blood flow in the hyperpermeable choriocapillaris. An uncontrolled case can be treated by a beta-adrenergic inhibitor such as metoprolol or metipranolol. Nonselective beta blockers can also be used. A recent experiment in animals shows that alpha-adrenergic inhibitors are somewhat more effective than beta-adrenergic inhibitors. Systemic acetazolamide helps in the resorption of sub-retinal fluid in CSC. It also promotes the healing of lesions of RPE, along with the preservation of function of vision in the long term. Transpupillary thermotherapy may be useful as it accelerates the CSC resolution, but its efficacy and long-term safety regarding the same are not yet known completely.

## Review

CSC may be understood as a prototype cause of the serous neuroretinal separation, which involves the macula. The clinical features of CSC suggest the contact loss between the RPE (a relative scotoma) and the photoreceptors and the foveal retina's bullous distension (micropsia and metamorphopsia). The detachment of the serous layer is because of lesions in the focal region and leakage (pathological) from the choriocapillaris through RPE. Although there are a lot of controversies regarding the role of stress, it would definitely be a good approach to counsel the CSC patient about the possible relation of stress from the very first visit. Serotonin concentrations in blood may influence the pathophysiology and, thereby, the prognosis of CSC [11].

If a patient shows current evidence of CSC that is chronic despite a short, reported symptom duration, it could be made out that there is asymptomatic detachment which may have been there for a longer time than the patient had been aware of it. Patients generally avoid unilateral symptom presentation. In comparison with reduced acuity of vision, subfoveal retinal pigment epithelial detachment (RPED) presents mainly as dysmorphia [12]. SRF typical for acute CSC in its short presentation results in poor outcomes as far as vision is concerned as it may adversely affect retinal morphology and function [13]. In CSC, whose onset is acute, a retinal laser does commonly have good results in eliminating or reducing leakage of RPE and thus helps in serous neuroretinal detachment's resolution. The prompt treatment benefit includes early resolution of SRF and, in the long term, possibly reduced recurrences and less degeneration of the RPE. Photocoagulation, when used, should be done so with proper care as it could induce subretinal neovascularization, even when primary treatment was given. Chronic CSC, commonly termed diffuse retinal pigment epitheliopathy, displays a pattern that is more complex in terms of RPE abnormalities. Indeed, usually in chronic detachment, there is irregular RPE hyper- and hypopigmentation, which may be the consequence of the atrophy of the photoreceptor.

The photocoagulation effect on chronic CSC is valid to a less extent than in acute CSC. PDT could be done many times in choroidal neovascularization (CNV) but repeated PDT is needed rarely in chronic CSC without CNV. In CSC, the safety and long-term efficacy of PDT have not been properly documented to date, and the description of the same is controversial. Systemic medications, although used, have no clear-cut role in the management of CSC. The exact mechanism behind the fovea's preferential detachment by the SRF is not known. As the fovea is thinner when compared to the rest of the macula, higher conductivity (hydraulic) could contribute to making the foveal retina less susceptible to the RPE's suction force and thus is more loosely attached with respect to the surrounding retina [14]. Advances in technology such as OCT and ICGA have led to a more clear-cut understanding of the pathophysiology of the disease. The pathogenesis of CSC is dependent on multiple factors and hence is a challenging task to understand [15]. One of the established complications of chronic CSC is silent type 1 CNV [16]. Unlike other imaging modalities, OCTA more frequently detected CNV [17]. There is widespread use of ICGA, which accepts choroidal disease in CSC [18].

A recurrent episode of SRF leakage develops in approximately one-third of CSC cases [19]. There are reports of retinal dragging with fibrin at early stages in eyes with acute CSC [20]. Modifications of PDT have changed treatment modalities of CSC. The ideal treatment modality is yet to be figured out in CSC [21]. The presence of atrophy of the foveal photoreceptor despite the successful reattachment after a symptomatic duration of around four months suggests that the active treatment should likely be commenced when the duration exceeds three months or so, provided that it would take some time to bring about the reattachment. PDT is successful in stopping the fluorescein without recurrence of CSC [22]. Half-dose verteporfin as PDT is effective in treating acute CSC cases that are symptomatic [23]. A 50% dose of verteporfin, when compared to a 30% dose, may be more effective in resolving fluorescein and SRF leakage [24]. Both half-fluence and half-dose PDT modifications were found to be effective similarly in improving the anatomic parameters, SRF, and visual acuity for chronic CSC [25,26]. Half-fluence PDT with verteporfin guided by ICGA helps treat acute CSC that is symptomatic [27]. Half-fluence PDT seems to be a good treatment option in patients who develop acute CSC while under systemic or local corticosteroid therapy for intraocular inflammatory diseases [28].

During OCT, one of the most important changes detected in the anatomy is a thinning of the outer nuclear layer [29]. In chronic CSC that is associated with fovea-involving PED and SRD, reduced-fluence PDT appears as a good treatment option [30]. Newer treatments in the form of mineralocorticoid receptor (MR) antagonists and anti-vascular endothelial growth factor appear to be promising to a great extent but need more clinical trials before implementing it in the management of CSC [31]. Treatment of CSC with half-dose verteporfin PDT significantly made the visual acuity better [32]. Treatment of CSC with oral medications like rifampicin, methotrexate (MTX), anti-androgenic drugs, and melatonin is under investigation [33]. There are clues hinting that bevacizumab seems effective in improving the visual and anatomical functions, and thus visual outcomes are improved in patients with CSC, but more evidence is required [34]. Transpupillary thermal therapy and MR antagonists are promising candidates for the treatment of CSC, but their safety profile and long-term efficacy need to be found through more research [33]. Higher availability and fewer adverse effects make subthreshold laser photocoagulation a relatively better treatment option for the management of CSC [35]. Once CSC is resolved, still in the near future, the patient may have ocular issues as a result of retinal thinning and choroidal flow defects [36].

## Conclusions

Advancements in medical science and technology have helped us gain more knowledge about various aspects of the clinical presentation and management of CSC. Newer imaging tools and techniques have given us a better insight into the disease and hence the management of the same. OCT with increased depth imaging, fluorescein angiography, fundus autofluorescence, angiography, and ICGA are all multimodal approaches to imaging for CSC. These multimodal imaging approaches have helped us attain great knowledge about this disease which was otherwise not very well known. Newer imaging approaches, along with the traditional modalities of imaging, have transformed our understanding of CSC, its pathophysiology, as well as diagnosis and management. As a result, the present approach to CSC management is greatly improving and refining, resulting in a better overall disease outcome. In the near future, definitely more research has to be carried out in order to effectively and comprehensively understand the exact whereabouts of CSC, and thus, ultimately, these would aid in its better management strategies.
